# Clinical correlates of physical performance and sarcopenia in Parkinson's disease: a cross-sectional study

**DOI:** 10.1055/s-0046-1816034

**Published:** 2026-02-27

**Authors:** Samuel Brito de Almeida, Danielle Pessoa Lima, João Rafael Gomes de Luna, Antonio Brazil Viana Júnior, Jarbas de Sá Roriz-Filho, Átila Pereira Alencar, Walter Oliveira Rios-Júnior, Wendel Carvalho de Oliveira, Pedro Lucas Grangeiro de Sá Barreto Lima, Paulo Ribeiro Nóbrega, Renan Magalhaes Montenegro-Júnior, Pedro Braga-Neto

**Affiliations:** 1 Universidade Federal do Ceará, Hospital Univerisátio Walter Cantídio, Unidade de Pesquisa Clínica, Fortaleza CE, Brazil.; 2 Universidade Federal do Ceará, Faculdade de Medicina, Departamento de Clínica Médica, Divisão de Geriatria, Fortaleza CE, Brazil.; 3 Universidade de Fortaleza, Faculdade de Medicina, Fortaleza CE, Brazil.; 4 Universidade Federal do Ceará, Faculdade de Medicina, Fortaleza CE, Brazil.; 5 Universidade Federal do Ceará, Faculdade de Medicina, Departamento de Clínica Médica, Divisão de Neurologia, Fortaleza CE, Brazil.; 6 Universidade Estadual do Ceará, Centro de Ciências da Saúde, Fortaleza CE, Brazil.

**Keywords:** Hand Strength, Parkinson Disease, Absorptiometry, Photon, Physical Functional Performance

## Abstract

**Background:**

Parkinson's disease (PD) presents motor and non-motor symptoms that impair function and quality of life. Identifying clinical factors linked to physical performance is key for patient care and management.

**Objective:**

To examine associations between sarcopenia-related measures and physical performance in mild-to-moderate PD (Hoehn & Yahr [HY] I–III).

**Methods:**

This was a cross-sectional study including patients with idiopathic Parkinson's disease at mild to moderate stages (Hoehn & Yahr I–III), evaluated in the ON medication state. Physical performance was assessed using the Short Physical Performance Battery (SPPB). Sarcopenia was evaluated according to the revised European Working Group on Sarcopenia in Older People (EWGSOP2) consensus, including screening with the SARC-F questionnaire and the Ishii score, assessment of muscle strength by handgrip dynamometry, and evaluation of body composition and appendicular lean mass by whole-body dual-energy X-ray absorptiometry (DXA). Analyses included bivariate comparisons, correlation analyses, and logistic regression models (Enter and Best Subsets).

**Results:**

A total of 127 patients were evaluated (mean age 66 years; 41.7% females). Low physical performance was observed in 39% (n = 50) of patients and was strongly associated with positive screening of sarcopenia (SARC-F score ≥ 4; odds ratio [OR]: 1.67; 95%CI: 1.30–2.15; *p* < 0.001). Ishii score ( *p* = 0.009), reduced mean handgrip strength (26 ± 10 kgf versus 30 ± 10 kgf; *p* = 0.02), and postural instability and gait difficulty (PIGD) ( *p* < 0.001) were also significantly associated with low SPPB performance in bivariate analyses. In the multivariable models, SARC-F and PIGD emerged as independent predictors of poor physical performance. The best subset model, combining SARC-F and PIGD, showed good discriminative accuracy (area under the curve [AUC] = 0.82).

**Conclusion:**

Higher PIGD scores and SARC-F ≥ 4 correlated with poor physical performance in PD. Low performance was linked to both SARC-F and Ishii scores, which help identify risk of functional decline. Longitudinal studies are needed to clarify causality and treatment implications.

## INTRODUCTION

Parkinson's disease (PD) impairs physical function and quality of life through its well-defined motor and non-motor symptoms. 1 While motor symptoms from the core PD motor dysfunction (bradykinesia, rigidity, and tremor) drive physical limitations, an important contributor to disability in PD is sarcopenia—the progressive loss of muscle mass and strength. 2 3 Evidence suggests muscle weakness may precede motor symptoms and influence disease progression. 4 Understanding physical performance and sarcopenia in PD could inform targeted interventions. Identifying clinical factors linked to these impairments supports comprehensive care strategies. A holistic approach addressing motor and non-motor contributors is crucial to optimizing outcomes. 5 No prior studies have examined whether physical performance scores correlate with sarcopenia and PD severity. The present study investigates associations between low physical performance, sarcopenia, and clinical outcomes in PD.

## METHODS

### Study participants

The present cross-sectional study was conducted at Hospital Universitário Walter Cantídio (HUWC), Fortaleza, Brazil, from May 2021 to April 2022. Participants were PD patients from a movement disorders outpatient clinic. Diagnosis followed the Movement Disorders Society (MDS) criteria. 6 Each patient was evaluated by two neurologists and one geriatrician.

Eligible participants had a confirmed PD diagnosis (Hoehn & Yahr [HY] stage 1–3), were at least 40 years old, and could stand and walk unassisted. Patients with HY stage 4 to 5 were excluded, as they could not complete the Five Times Sit-to-Stand Test (FTST), balance, and gait speed tests. Individuals with severe health conditions or uncontrolled chronic diseases affecting safety or data interpretation were excluded. These conditions included New York Health Association (NYHA) class III to IV heart failure, dialysis-dependent renal disease, severe chronic obstructive pulmonary disease (COPD), advanced osteoarthritis, active cancer (except localized prostate/skin cancer), and moderate-to-severe dementia (Clinical Dementia Rating [CDR] 2–3).

Patients were also excluded if they had recent contrast/radionuclide exposure (72 hours), pregnancy, deep brain stimulation, or pacemakers, as these factors could interfere with dual energy x-ray absorptiometry (DEXA). All participants provided written informed consent, and the study was approved by the Research Ethics Committee (register no. 91075318.1.0000.5045). The study adhered to the Declaration of Helsinki guidelines.

### Clinical evaluation

A structured interview was conducted to collect medical and sociodemographic data. Dementia was defined according to DSM-5 criteria and osteoporosis according to the National Osteoporosis Foundation guidelines. 7 Data on antiparkinsonian medication use, including levodopa (L-dopa), amantadine, dopamine agonists, catechol-O-methyltransferase (COMT) inhibitors, monoamine oxidase type B (MAO-B) inhibitors, and different L-dopa formulations, were collected. The levodopa equivalent dose (LED) was calculated following Tomlinson et al. 8

Functional assessments included the Schwab and England Activities of Daily Living Scale 9 for daily activities and modified HY staging 10 for disease severity. Motor function was evaluated using the Movement Disorder Society-Sponsored Revision of the Unified Parkinson's Disease Rating Scale Part III (MDS-UPDRS III), 11 and postural instability was assessed with the Postural Instability Gait Disorder (PIGD) score, derived from UPDRS III items 3.9 (sit-to-stand), 3.10 (gait), 3.12 (postural stability), and 3.13 (posture). Lower limb bradykinesia (LLB) was calculated using UPDRS-III items 3.7 (toe tapping), 3.8 (leg agility), and 3.9 (arising from a chair). Cognition was assessed with the Mini-Mental State Examination (MMSE), and depressive symptoms were evaluated using the 15-item Geriatric Depression Scale (GDS-15). 12

Falls were defined as unintentional ground-level events, excluding those caused by seizures, accidents, or syncope. Patients were questioned about falls occurring in the previous 1 and 6 months, and responses were cross-checked with caregivers and clinical records. Self-reported physical activity was assessed during the interview through the question: 'Do you practice physical activity at least 3 times per week for a minimum of 30 minutes per session?' Although current guidelines usually recommend 150 minutes per week, 11 given that our study involved a frailer population, we chose a lower cutoff in order to increase sensitivity and better discriminate between sedentary individuals and those engaging in at least moderate levels of physical activity.

### Sarcopenia and physical performance assessment

Calf circumference (CC) was measured at the widest point of the right calf in a seated position. 13 Probable sarcopenia was defined as low handgrip strength, while confirmed sarcopenia was diagnosed per the European Working Group on Sarcopenia in Older People (EWGSOP2) criteria as low handgrip strength and low appendicular skeletal muscle mass. 14 Severe sarcopenia was identified when low muscle strength, reduced muscle quantity/quality, and impaired physical performance were all present.

We used the SARC-F as a screening tool to assess the likelihood of sarcopenia. The instrument consists of five simple and rapid questions addressing handgrip strength, ability to climb a flight of stairs, walking between rooms, rising from a chair, and history of falls in the previous year. The Ishii Test, a rapid screening tool, estimated sarcopenia probability based on age, handgrip strength, and CC, using cutoff values of ≥ 105 for men and ≥ 120 for women. 15

Handgrip strength was measured using a SAEHAN dynamometer (SAEHAN Corporation), following the Southampton protocol, with a cutoff of < 27 kg for men and < 16 kg for women. 16 Participants performed three trials per hand, and the highest value was recorded.

Physical performance was evaluated using the Short Physical Performance Battery (SPPB), which includes balance, gait speed, and lower limb strength assessments: 17 balance test (participants maintained three progressively difficult stances as follows feet together, semi-tandem, and full tandem for 10 seconds each); gait speed test (recorded over 4 meters); chair stand test (five sit-to-stand repetitions).

Short Physical Performance Battery scores ranged from 0 to 12. Low physical performance was defined as an SPPB score ≤ 8 points, in accordance with the recommendation of the EWGSOP2; 14 this cutoff is frequently used as a pragmatic screening threshold and has been associated with higher risk of adverse outcomes, including cardiovascular events and mortality. 18 19 Appendicular skeletal muscle mass (ASMM) was assessed via DEXA, and lean mass index (LMI) was calculated by dividing ASMM by height squared. Low muscle mass was defined as LMI < 7 kg/m ^2^ (men) and < 5.5 kg/m ^2^ (women) per EWGSOP2.

All patients underwent handgrip strength, MMSE, SPPB, GDS-15, UPDRS III, and HY staging assessments in the “on” state.

### Statistical analysis

Data collection and management were performed using Research Electronic Data Capture (REDCap – Vanderbilt University). Patients were categorized as having adequate (SPPB ≥ 8) or low (SPPB < 8) physical performance.

Categorical variables were reported as frequencies (%), and continuous variables as mean ± standard deviation (median) values. Bivariate analyses included Fisher's exact test for categorical variables and Mann-Whitney U/Wilcoxon tests for non-normally distributed continuous variables. The Student's *t* -test was used for normally distributed continuous variables.

Multivariate logistic regression was conducted to analyze the impact of clinical and sarcopenia-related factors on physical performance:

- Model 1.a: Included sociodemographic and clinical variables.
- Model 1.b: Included sarcopenia-related variables.
- Final model 1: Combined significant variables from Models 1.a and 1.b.
- Model 2: Identified key predictors of low vs. adequate performance using the Best Subsets regression method.

Spearman's correlation assessed relationships between clinical and sarcopenia-related factors and SPPB scores. A logistic regression model was applied to construct a receiver operating characteristic (ROC) curve, with statistical significance set at *p* < 0.05. Data analysis was performed using R software (R Foundation for Statistical Computing).

## RESULTS

The study included 129 patients with a mean age of 66 ± 11 years. Fifty-four (42.0%) were female. The most prevalent comorbidities were hypertension (47.0%), depression (31.0%), and dyslipidemia (16.0%). **Supplementary Material** (available at https://www.arquivosdeneuropsiquiatria.org/wp-content/uploads/2025/11/ANP-2025.0147-Supplementary-Material.docx ). **Table S1** summarizes clinical and demographic characteristics.

The mean SARC-F score was 4.07 ± 2.79, with 50% (n = 65) scoring ≥ 4, indicating sarcopenia risk. Low grip strength (< 27 kg for men, < 16 kg for women) was found in 20% (n = 26). The mean SPPB score for the cohort was 8.80 ± 2.61, with 50 patients (39%) classified as having low physical performance (SPPB ≤ 8). Confirmed sarcopenia (low strength and low muscle mass) was present in 10.2% of participants. Anthropometric and sarcopenia data are summarized in Table 1 . Table 2 presents the bivariate analysis for low physical performance based on SPPB scores. Table 3 displays correlation analyses between SPPB and various clinical and anthropometric variables.

**Table 1:** Anthropometric characteristics and components of sarcopenia of the sample

FEATURES		N = 129 1
**BMI (kg/m2)**		26.4 ± 4.4 (26.5)
**Calf circumference (cm)**		33.7 ± 3.6 (33.8)
**Total body fat**		32 ± 9 (33)
**SARC-F score**		4.07 ± 2.79 (4.00)
**SARC-F ≥ 4**		65 (50%)
**Grip strength**		28 ± 10 (28)
**FTST**		17 ± 8 (15)
**SPPB (subtest): Balance**		
• 0 - Unable		4 (3.1%)
• 1 - Able only to stand, feet together side by side, for 10 seconds		11 (8.5%)
• 2 - Score 1 plus able to stand heel of one foot against side of big		18 (14%)
• 3 - Score 2 plus able to stand feet aligned heel to toe for		15 (12%)
• 4 - Score 2 plus able to stand feet aligned heel to toe		81 (63%)
**SPPB (subtest): Gait speed**	• 0 - Failed to	2 (1.6%)
	• 1 - Time > 8.70 sec	4 (3.1%)
	• 2 - Time from 6.21–8.70 sec	9 (7.0%)
	• 3 - Time from 4.82–6.20 sec	10 (7.8%)
	• 4 - Time < 4.82 sec	104 (81%)
**SPPB (subtest): FTST**	• 0 - Failed or time > 60 sec	15 (12%)
	• 1 - Time of 16.70 sec or more	51 (40%)
	• 2 - Time from 13.70–16.69 sec	13 (10%)
	• 3 - Time from 11.20–13.69 sec	22 (17%)
	• 4 - Time of 11.19 sec or less	28 (22%)
**SPPB total score**		8.80 ± 2.61 (9.00)
**Average protein intake g/kg/day**		1.19 ± 0.40 (1.15)
**Confirmed sarcopenia**		13 (10%)
**ISHII summation score ≥ 120 in women and ≥ 105 in men**		46 (37%)
**Gait speed (m/s)**		1.35 ± 0.52 (1.37)
**SPPB interpretation**	• Adequate physical performance (> 8)	79 (61%)
	• Poor physical performance (≤ 8)	50 (39%)

Abbreviations: BMI, Body Mass Index; DEXA, dual-energy X-ray absorptiometry; FTST, five times sit-to-stand test; SPPB, Short Physical Performance Battery.

Notes: n (%); mean ± standard deviation (median).

**Table 2:** Bivariate analysis of the characteristics of the sociodemographic and clinical profile and components of sarcopenia and physical performance of patients with mild-to-moderate Parkinson's disease, Fortaleza, CE, from May 2021 to April 2022

Variables		Adequate physical performance (SPPB > 8), n = 79 1	Low physical performance (SPPB ≤ 8), n = 50 1	*p* -value
Sex	Female	31 (39%)	23 (46%)	0.448
	Male	48 (61%)	27 (54%)	
**Age**		64 ± 10 (63)	68 ± 11 (70)	**0.016**
**Marital status**	Married	57 (73%)	33 (66%)	0.433
	Single	8 (10%)	5 (10%)	
	Stable union	2 (2.6%)	2 (4.0%)	
	Widower	5 (6.4%)	8 (16%)	
	Divorced	6 (7.7%)	2 (4.0%)	
**Family history**		33 (43%)	20 (41%)	0.774
**Comorbidities**	Hypertension	37 (47%)	24 (48%)	0.897
	Type-2 diabetes	8 (10%)	7 (14%)	0.504
	Dyslipidemia	10 (13%)	11 (22%)	0.161
	Depression	21 (27%)	18 (37%)	0.243
**Number of medicines**		5.00 ± 2.09 (5.00)	5.84 ± 2.28 (6.00)	**0.038**
**Use of walking aid**		12 (15%)	15 (31%)	**0.041**
**Self-reported physical**		41 (53%)	12 (24%)	**0.002**
**Visual hallucinations**		11 (14%)	15 (31%)	**0.025**
**Dyskinesia**		39 (50%)	30 (61%)	0.216
**HY**	1	2 (2.6%)	0 (0%)	**< 0.001**
	1.5	0 (0%)	1 (2.0%)	
	2	21 (27%)	3 (6.1%)	
	2.5	36 (46%)	18 (37%)	
	3	19 (24%)	27 (55%)	
**Schwab and England**		87 ± 9 (90)	80 ± 14 (80)	**0.006**
**Freezing of gait**		30 (38%)	25 (51%)	0.164
**Number of falls**		2.33 ± 10.57 (0.00)	5.76 ± 25.80 (1.00)	**< 0.001**
**SARC-F score**		2.92 ± 2.31 (3.00)	5.88 ± 2.53 (6.00)	**< 0.001**
**SARC-F ≥ 4**		26 (33%)	39 (78%)	**< 0.001**
**Handgrip strength**		30 ± 10 (30)	26 ± 10 (26)	**0.020**
**BMI**		26.2 ± 4.2 (26.4)	26.8 ± 4.8 (26.8)	0.505
**Calf circumference**		34.0 ± 3.4 (33.5)	33.3 ± 3.9 (34.0)	0.456
**MDS-UPDRS part 3**		39 ± 13 (37)	51 ± 15 (55)	**< 0.001**
**Average protein intake**		1.20 ± 0.40 (1.19)	1.18 ± 0.41 (1.12)	0.675
**Postural hypotension**		15 (19%)	12 (24%)	0.495
**Constipation**		30 (38%)	27 (54%)	0.074
**MMSE**		24.8 ± 3.7 (26.0)	22.4 ± 4.9 (23.0)	**0.005**
**GDS-15**		4.36 ± 3.22 (4.00)	6.08 ± 3.54 (6.00)	**0.006**
**General bradykinesia**		14 ± 6 (13)	19 ± 8 (18)	**< 0.001**
**Upper bradykinesia**		8.6 ± 4.2 (9.0)	12.1 ± 5.2 (12.0)	**< 0.001**
**Lower bradykinesia**		5.3 ± 2.4 (5.0)	7.2 ± 3.6 (7.0)	**0.003**
**Equivalent levodopa**		10.6 ± 5.4 (10.6)	12.7 ± 6.0 (13.1)	**0.041**
**Confirmed sarcopenia**		9 (11%)	4 (8.0%)	0.533
**HY**	Mild	23 (29%)	4 (8.2%)	**0.004**
	Moderate	55 (71%)	45 (92%)	
**PIGD**		4.35 ± 2.19 (4.00)	6.96 ± 2.33 (7.00)	**< 0.001**
**Ishii score ≥ 120 in women and ≥ 105 in men**		21 (28%)	25 (51%)	**0.009**

Abbreviations: BMI, Body Mass Index; GDS-15 geriatric depression scale; HY, Hoehn & Yahr scale; MDS-UPDRS: unified Parkinson's disease rating scale; MMSE, Mini-Mental Status Examination; PD, Parkinson's disease; PIGD, Postural instability gait difficulty; SARC-F questionnaire, Five questions about Strength, Assistance with walking, Rise from a chair, Climb stairs and Falls. questionnaire.

**Table 3:** Correlation analysis between total and partial SPPB scores

Parameters	SPPB	SPPB balance	SPPB gait	SPPB strength
Age	**−0.270∗∗**	**−0.196∗**	**−0.312∗∗**	**−0.271∗∗**
Duration of PD disease	**−0.181∗**	−0.142	−0.079	−0.079
Number of comorbidities	−0.082	−0.092	−0.114	−0.092
Levodopa equivalent dose	−0.074	−0.105	0.01	0.019
MDS-UPDRS part 3	**−0.421∗∗**	**−0.264∗∗**	**−0.361∗∗**	**−0.343∗∗**
Overall bradykinesia	**−.493∗∗**	**−.322∗∗**	**−.419∗∗**	**−.416∗∗**
Lower limb bradykinesia	**−.262∗∗**	**−.190∗**	**−.291∗∗**	**−.203∗**
Upper bradykinesia subscore	**−.306∗∗**	**−.224∗**	**−.294∗∗**	**−.216∗**
PIGD subscore	**−.574∗∗**	**−.374∗∗**	**−.500∗∗**	**−.456∗∗**
HY scale	**−0.469∗∗**	**−0.425∗∗**	**−0.387∗∗**	**−0.252∗**
Schwab and England	**0.198∗**	0.156	**0.214∗**	0.132
MMSE	**0.390∗∗**	**0.277∗∗**	**0.336∗∗**	**0.317∗∗**
GDS-15	**−0.266∗∗**	**−0.180∗**	−0.113	−0.232∗∗
SARC-F	**−0.588∗∗**	**−0.356∗∗**	**−0.420∗∗**	**−0.468∗∗**
BMI	0.003	0.017	−0.023	−0.005
Right calf circumference	0.084	0.101	0.182∗	0.044
Handgrip strength	**0.395∗∗**	**0.179∗**	**0.427∗∗**	**0.373∗∗**
ALM/Height2	**0.211∗**	0.110	**0.219∗**	**0.222∗**
Falls in the last 6 months	**−0.326∗∗**	**−0.211∗**	**−0.212∗**	**−0.273∗∗**

Abbreviations: ALM, appendicular lean mass; BMI, body mass index; GDS-15 geriatric depression scale; HY, Hoehn & Yahr scale; LEDD, levodopa equivalent daily dose; MDS-UPDRS: unified Parkinson's disease rating scale; MMSE, Mini-Mental Status Examination; PD, Parkinson's disease; SARC-F questionnaire, Five questions about Strength, Assistance with walking, Rise from a chair, Climb stairs and Falls; SPPB, Short Physical Performance Battery.

Notes: Correlations are displayed using Spearman's rho (ρ). *Correlation is significant at level 0.05 (two-tailed). **Correlation is significant at level 0.01 (two-tailed).

Two regression models were applied. In the ENTER model, three regression analyses were conducted: the first including sociodemographic and clinical variables, the second incorporating sarcopenia-related variables, and the third selecting variables significantly associated with physical performance in the prior analyses. The Best Subset model performed a single regression analysis.

A Spearman correlation analysis examined relationships between bradykinesia parameters and UPDRS III. Due to multicollinearity, upper and lower limbs' bradykinesia parameters were removed from subsequent analyses, and MDS-UPDRS III was retained in the ENTER logistic regression model. This decision was based on the strong correlations observed between general, upper, and lower limb bradykinesia and the UPDRS III total score, as well as the high variance inflation factor (VIF) values.

**Supplementary Material Table S2** presents the logistic regression results for clinical variables (model 1a), in which only the PIGD score was significantly associated with physical performance. **Supplementary Material Table S3** shows the logistic regression results for sarcopenia-related variables (model 2a), in which sarcopenia screening was the only significant predictor. Table 4 presents the final model 1 logistic regression results.

**Table 4:** Enter regression analysis (final model 1) of variables associated with physical performance in patients with PD

Features	N	OR _1_	95%CI ^1^	*p* -value	VIF
**PIGD**	127	1.53	1.25, 1.94	**< 0.001**	1.0
**Sarcopenia screening**	127	3.97	1.63, 10.0	**0.003**	1.0

Abbreviations: OR, odds ratio; VIF, variance inflation factor; PIGD, postural instability and gait difficulty.

The Best Subset regression analysis evaluated combinations of independent variables, including PIGD, sarcopenia screening, physical activity, age, use of walking aid, lower limb bradykinesia, handgrip strength, and ISHII score. The Akaike Information Criterion (AIC) was used to identify the best model for discriminating adequate vs. low physical performance in PD patients. Among 9 models evaluated, SARC-F ≥ 4 and PIGD yielded the lowest AIC value, indicating the best discrimination model ( Table 5 ).

**Table 5:** Comparison of regression models created by the best subset regression method (model 3) using the Akaike Information Criterion (AIC)

Model - independent variables	AIC	R-square	Adj. R-square
1-PIGD	144.4938	0.2515	0.2454
2- Sarcopenia screening + PIGD	**135.4966**	0.3151	**0.3037**
3- Physical activity + sarcopenia screening + PIGD	135.5733	0.3256	0.3087
4 - Physical activity + sarcopenia screening + PIGD + age	136.4268	0.3318	0.3093
5- Physical activity + sarcopenia screening + PIGD + age + use of walking aid	138.2938	0.3325	0.3042
6- Physical activity + sarcopenia screening + PIGD + age + use of walking aid + lower limb bradykinesia	140.2372	0.3328	0.2986
7- Physical activity + sarcopenia screening + PIGD + age + use of walking aid + palm pressure measurement + Ishii score (PC: 1,148)	142.1621	0.3332	0.293
8- Physical activity + sarcopenia screening + PIGD + age + use of walking aid + lower limb bradykinesia + palm pressure measurement + Ishii score (PC: 114 ♂ )	144.1281	0.3334	0.287
9 - Physical activity + sarcopenia screening + PIGD + age + use of walking aid + lower limb bradykinesia + palm pressure measurement + Ishii score (PC: 114 ♂ ) + HY	146.1278	0.3334	0.2808

Abbreviations: HY, Hoehn & Yahr scale; PC,; PIGD, postural instability and gait difficulty.

**Figure 1:**
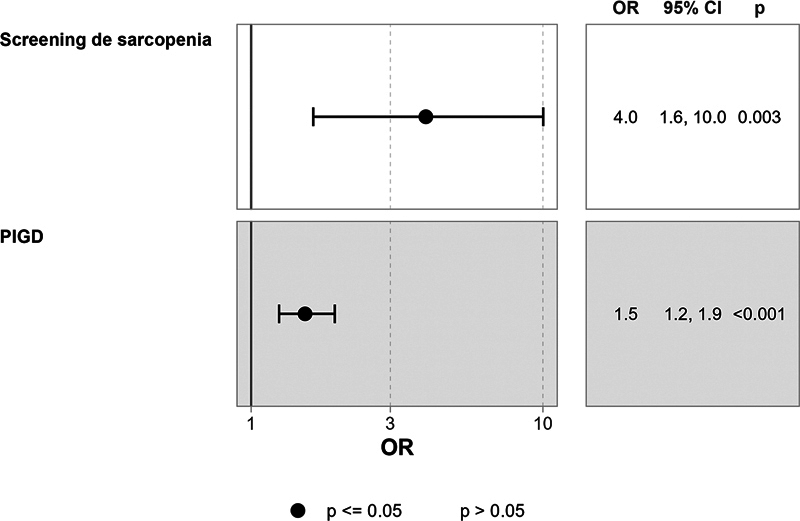
odds ratio of variables in the optimal model identified by Best Subset regress.

**Figure 2:**
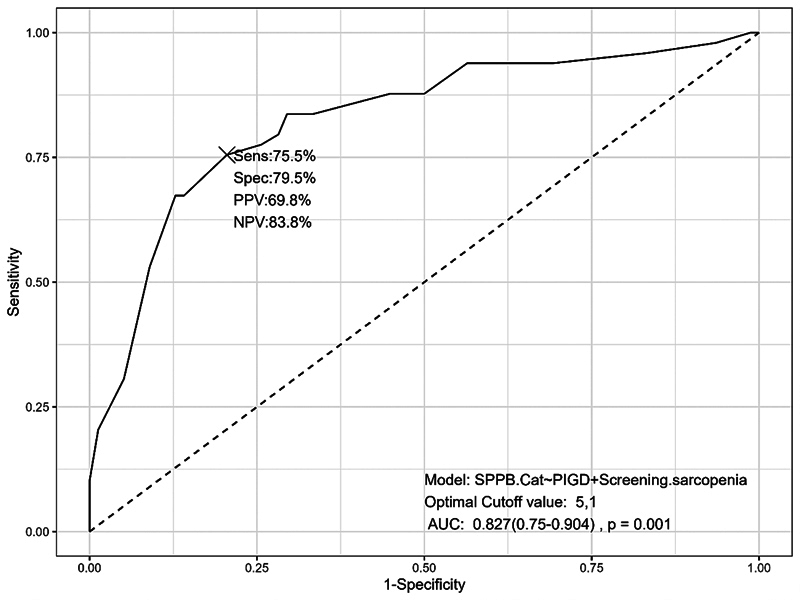
Analysis of accuracy, sensitivity, specificity, positive predictive value, negative predictive value, optimal cut-off point, and area under the curve of the optimal model discriminating low and adequate physical performance.

Figure 1 presents odds ratios for key variables identified in the Best Subset regression model, illustrating the strength of associations with physical performance. Figure 2 displays the ROC curve, along with sensitivity, specificity, accuracy, positive predictive value (PPV), and negative predictive value (NPV) for the optimal model. The ROC curve (area under the curve [AUC] = 0.82) demonstrated good predictive accuracy, with an optimal cutoff of 5.1 to maximize discrimination between adequate and low physical performance.

## DISCUSSION

The present study aimed to identify the clinical and sarcopenia-related factors associated with physical performance in patients with mild-to-moderate PD. Our primary finding is that a simple sarcopenia screening questionnaire (SARC-F) and a clinical measure of postural and gait instability (PIGD score) were the most powerful independent predictors of low physical performance.

The SARC-F is a widely used screening tool for identifying older adults with reduced physical function. The questionnaire uses upper limb strength, need for walking assistance, difficulty rising from chairs and climbing stairs, and history of falls to assess risk of sarcopenia. 20 In addition, it has been described as a factor associated with falls 21 and disability 13 in people with PD. 20

However, SARC-F has some limitations that should be considered. The SARC-F does not consider physiological and metabolic variables that can influence sarcopenia, such as detailed body composition and physical activity levels.

Originally designed as a screening tool for sarcopenia, the accuracy of the SARC-F questionnaire in patients with PD remains uncertain. A key concern in PD is that its questions (e.g., difficulty rising from a chair, history of falls) may overlap with parkinsonian motor symptoms rather than sarcopenia itself. 13 To minimize this potential bias, all participants were evaluated in the “on” state under L-dopa treatment.

However, in our multivariable model, SARC-F emerged as an independent predictor even after accounting for the PIGD score. This suggests that SARC-F captures a broader construct related to frailty and functional decline that is distinct from the motor impairments measured by the PIGD score alone. Also, it directly assesses the occurrence of falls over the past year, a feature that is highly specific for predicting future falls.

The PIGD score, which reflects axial motor dysfunction, was also a strong, independent predictor. This is expected, as postural instability and gait difficulty are cardinal features of advancing PD that directly lead to reduced mobility, increased fall risk, and loss of autonomy. 22 23 As PD progresses, the impact on quality of life is significant, as physical limitation can lead to social isolation, reduction of physical activity and increased dependence. 13 Our finding aligns with robust evidence showing that motor symptoms are a primary driver of physical impairment in PD, and regular exercise may provide significant improvements in muscle strength, balance, mobility, and risk of falls in PD patients. 24

We also observed that low physical performance was associated with lower self-reported physical activity. This is consistent with existing literature that shows that sedentary patients with PD had poorer balance, lower functional mobility, and were more likely to have fallen in the previous year. 25

The Ishii score demonstrated a significant association with physical performance measured by the SPPB ( *p* = 0.009). Patients with higher scores on the Ishii test tend to perform worse on the SPPB, 26 which suggests that the score may be a good indicator of functional decline in individuals with PD. In our bivariate analysis, both the Ishii score and the SARC-F were significantly associated with physical performance, but only the SARC-F remained an independent predictor in the multivariable models. Taken together, these findings suggest that while the SARC-F may serve as a pragmatic and robust screening tool and the Ishii score as a provider of complementary information that is more closely linked to objective sarcopenia parameters, particularly valuable in contexts where direct measures of muscle mass are not feasible.

Our study associated physical performance with the MDS-UPDRS III score, as well as with the subscores of body bradykinesia, upper limb bradykinesia, lower limb bradykinesia, and postural instability. Previous studies have shown that motor symptoms can impact physical performance in PD. 27 Luz et al. 28 found a statistically significant association between the SARC-F questionnaire and MDS-UPDRS II scores, but not with MDS-UPDRS III scores. However, the same study observed that individuals with PD and sarcopenia generally obtained higher scores (indicating worse conditions) in sections II, III, and II + III compared to those without sarcopenia. The lack of statistical significance in these results may be attributable to the relatively small number of participants with PD and sarcopenia (n = 15) compared to non-sarcopenic participants (n = 62). A recent systematic review demonstrated that muscle strength is associated with functional capacity, disease severity, and parkinsonian symptoms measured by MDS-UPDRS score. 29

It should also be acknowledged that patients with more advanced PD—reflected by higher HY stages, poorer cognitive/mood status, and greater bradykinesia—were overrepresented among those with low SPPB scores. Disease severity may have contributed to the associations observed. Our analytical strategy incorporated several robust approaches to address this concern, including bivariate analyses, correlation analyses, and multivariable logistic regressions using both ENTER and Best Subsets methods, while carefully managing multicollinearity. Across these models, SARC-F and PIGD consistently emerged as independent predictors of low physical performance, and the final model demonstrated good discriminative accuracy (AUC = 0.82). These findings suggest that the associations we report are not solely attributable to the predominance of more severe cases in the lowest-performing group even though residual confounding by disease severity cannot be excluded.

Another relevant finding in our study was the higher frequency of falls among patients with poor physical performance (SPPB ≤ 8). This highlights that the observation of reduced physical performance may also serve as a warning sign for increased fall risk in PD. The incorporation of SPPB into routine practice may help identify patients at higher risk and guide the implementation of preventive interventions.

Research on sarcopenia in individuals with PD reveals a significant disparity in prevalence rates, ranging from 6 to 55.8% as indicated in several studies. 30 31 This variation can be attributed to methodological differences and a lack of consensus on definitions of sarcopenia. 30 A particular challenge is the absence of validated criteria for the diagnosis of sarcopenia in PD patients. 32 This diversity in definitions results in a wide range of clinical interpretations, which may complicate the therapeutic approach for sarcopenia.

Allen et al. 33 investigated the relationship between muscle weakness and bradykinesia in patients with PD. The study compared the strength and leg extensors muscle power of 40 PD patients with a control group of 40 healthy individuals. The results showed that PD patients had a 172 N lower force and a 124 W lower power at peak compared to controls. Bradykinesia was evident in light to medium loads, but not in heavy loads. Thus, muscle weakness contributed to the reduction of power at all loads, while bradykinesia influenced only the lighter loads, suggesting a combination of weakness and bradykinesia at light loads and only weakness at heavy loads. 30 Several studies suggest that patients with PD are at increased risk of sarcopenia compared to the general population; however, the literature is limited by methodological variability and differing diagnostic criteria. 34

Our data also showed that patients with higher physical performance (SPPB > 8) had a lower mean age than patients with low physical performance (68 ± 12 versus 64 ± 10; *p* = 0.016). The findings of the present study agree with the current literature, which consistently points to age as a critical determinant of physical performance, particularly in the context of PD. 35

In the present study, 33% of the participants with poor physical performance had visual hallucinations, while only 13.4% of the participants with good performance had visual hallucinations ( *p* < 0.008). These results can be expected, as individuals who present visual hallucinations are typically classified into moderate to severe stages, associated with cognitive dysfunctions and postural instability. 36

Cognitive function (measured by MMSE) was significantly lower in individuals with low physical performance compared to those with higher performance as shown in other studies. 37 Similarly, individuals with higher depressive symptom scores on GDS-15 had worse physical performance compared to those with milder depressive symptoms, a finding previously reported by other authors. 38

The present study has several limitations. The research sample was recruited from a single tertiary center, which may limit generalizability. We only included patients with mild-to-moderate PD (HY 1-3) and used SPPB cut-off points developed for the general elderly population, 14 as PD-specific values are not yet established. In addition, data on the prevalence of poor physical performance and its determinant variables in people with PD could not be compared with a control group.

Despite these limitations, our study has significant strengths. We identified an association between poor physical performance and two simple, low-cost clinical tools—the SARC-F and the PIGD score—that can be easily implemented in routine practice. The final predictive model combining these two variables showed good discriminative accuracy (AUC = 0.82). The present study contributes to the existing literature by identifying a combination of variables that can improve the assessment of physical performance in patients with PD.

The results highlight a multidimensional assessment of sarcopenia, PIGD in PD. The inclusion of sarcopenia screening and PIGD in clinical evaluation strengthens the ability of healthcare professionals to identify and treat patients with compromised physical performance.
